# Vermicomposting Reshapes Microbial Communities and Functional Potential in Sewage Sludge: Insights from 16S and ITS Metabarcoding

**DOI:** 10.3390/biotech15040083

**Published:** 2026-09-25

**Authors:** Isabel Berdecio, Manuel Aira, Marcos Pérez-Losada, Jorge Domínguez

**Affiliations:** 1 Computational Biology Institute, Department of Biostatistics and Bioinformatics, Milken Institute School of Public Health, George Washington University, Washington, DC 20052, USA; iberdecio3@gwu.edu (I.B.); mlosada@gwu.edu (M.P.-L.); 2 Grupo de Ecoloxía Animal (GEA), Universidade de Vigo, E-36310 Vigo, Spain; jdguez@uvigo.es

**Keywords:** antibiotic resistance, antifungal resistance, biosolid, earthworm, human pathogen, sewage sludge, sustainability, vermicompost

## Abstract

Vermicomposting has been shown to be a cost-effective and sustainable approach for improving the environmental safety of sewage sludge, yet it remains comparatively understudied at the industrial scale. This study investigates how vermicomposting with the earthworm *Eisenia andrei* alters the taxonomic and functional diversity of bacterial and fungal communities in sewage sludge, including human-associated pathogens. Using 16S rRNA and ITS metabarcoding, we compared fresh sludge and sludge aged for 13 months without earthworms to vermicomposted sludge after 13 months. The study included one pilot-scale vermireactor and one control pilot-scale reactor with ten spatial subsamples per sampled layer (30 subsamples). Bioinformatic and statistical analyses showed that vermicomposting may produce significant shifts in taxonomic composition, reduce the abundance of baseline taxa, and enrich decomposer-associated bacteria and fungi. Both bacterial and fungal alpha diversity (e.g., Shannon) increased over time, while beta diversity (e.g., Bray–Curtis) analyses indicated heterogeneous changes in community structure across treatments and time points. Vermicomposting also seemed to activate the prediction of pathways associated with nitrogen metabolism, amino acid biosynthesis, and pollutant degradation, while reducing the prevalence of predicted antibiotic resistance. Human bacterial pathogens exhibited persistence but also temporal variation, suggesting compositional shifts during vermicomposting. Fungal communities transitioned from pathogen-associated taxa to predominantly saprotrophic and multifunctional groups, particularly in vermicompost. Overall, these findings suggest that vermicomposting may contribute to restructuring microbial communities in sewage sludge, with potential implications for the sustainability and safety of this biosolid.

## 1. Introduction

Sewage sludge is a semi-solid byproduct generated during wastewater treatment. Primary sludge is produced through the settling of suspended solids and organic matter from wastewater. Secondary (biological) sludge is generated during biological treatment processes, in which microorganisms consume dissolved organic matter, multiply, and form dense flocs that are subsequently separated during the secondary settling stage. The resulting sludge is then dewatered and stabilized. This treated biosolid is widely used for land applications, including agriculture, land reclamation, soil restoration, forestry, and domestic landscaping such as lawns and gardens [1,2]. The use of biosolids in land application promotes sustainability by returning organic matter to the soil and providing economic benefits to farmers by acting as a substitute for purchased fertilizers [1,3]. Increased urbanization has led to a rise in sewage sludge production [4,5], which poses environmental and health risks due to the presence of pathogens—such as bacteria (*Legionella*, *Yersinia*, and *Escherichia coli*) and fungi (*Aspergillus*, *Candida*, and *Rhizopus*)—as well as pharmaceuticals, chemicals (e.g., toxic trace elements such as selenium, titanium, and silver), and pesticides present in the sludge [6,7]. Moreover, the use of biosolids may promote the spread of antibiotic-resistant genes (ARGs), with studies highlighting their role in facilitating gene transfer across microbiomes and environmental compartments [4,8,9]. Similarly, antifungal resistance has emerged as a growing concern, as the extensive use of fungicides in agriculture contributes to its development and dissemination in the environment [4,10].

Vermicomposting is an aerobic process by which earthworms and other microorganisms stimulate decomposition and biostabilization by modifying the chemical, physical, and microbiological properties of organic waste [11,12]. Vermicomposting consists of two main phases: an active phase and a maturation phase. The active phase encompasses processes associated with digestion in the earthworm gut, collectively referred to as gut-associated processes. During the maturation phase, earthworms excrete feces (casts), which undergo aging and microbial community turnover; these are termed cast-associated processes [13]. Vermicomposting is a readily implementable, cost-effective, and environmentally sustainable technique to transform sewage sludge [13,14]. Previous research has shown that vermicomposting can reduce or even eliminate more than 90% of bacterial and fungal taxa [13], including human-associated bacterial and fungal pathogens [12], remove 81–90% of pharmaceuticals and chemicals [15], and attenuate ARGs load [16,17], demonstrating its overall effectiveness. However, pathogen levels in sewage sludge may decline at variable rates over time [18,19], while ARGs can persist or fluctuate, making spontaneous decay an unreliable sanitation method [20,21,22] and potentially increasing health risks associated with land application. Previous studies evaluating pathogen and ARG reductions during sewage sludge vermicomposting have primarily examined relatively short periods of treatment of approximately 3 months [12,16]. A key research gap remains in understanding the microbial effects of long-term vermicomposting of sewage sludge, particularly compared with natural maturation in the absence of earthworms. Distinguishing changes attributable to vermicomposting from those resulting from maturation requires a control treatment maintained under comparable conditions. However, such controls have generally been lacking in previous studies. Furthermore, microbial responses to vermicomposting under operationally relevant, continuous-feeding conditions remain poorly characterized. Therefore, it is important to determine the specific contributions of vermicomposting beyond those of natural maturation and to evaluate the distinct environmental benefits associated with this treatment.

Accordingly, this study aimed to compare the effects of vermicomposting and natural maturation of sewage sludge on bacterial and fungal taxonomic composition, diversity, pathogen abundance, and predicted functional pathways. Comparisons were conducted across three substrates: initial sewage sludge, sewage sludge aged for 13 months without earthworms, and 13-month-old vermicompost produced at industrial scale. In pursuit of these aims, we used 16S rRNA and ITS metabarcoding to characterize bacterial and fungal communities, respectively. We hypothesized that vermicomposting would promote the assembly of bacterial and fungal communities distinct from those arising through natural maturation alone, characterized by shifts in microbial diversity and community composition, reductions in potential pathogen abundance, and enrichment of functional pathways associated with organic matter decomposition and nutrient transformation.

## 2. Materials and Methods

### 2.1. Experimental Design and Sampling

Sewage sludge (SS) was collected from a wastewater treatment plant (WWTP) located in Moaña. A pilot-scale vermireactor (5.8 m × 1.45 m × 1 m) with a capacity of 4.5 m^3^ was established. The vermireactor was initially prepared with a 10 cm layer of vermicompost to provide shelter for the earthworms and stocked with an initial density of 3000 individuals m^−2^ of the earthworm *Eisenia andrei* (Bouché, 1972). New layers of fresh SS (250 kg) were added weekly for two years. In one corner of the vermireactor, a plastic mesh was placed between layers to separate each SS addition. This protocol enabled precise dating and sampling of each vermicomposting layer. Ten subsamples of the initial fresh SS were collected and stored at −80 °C. A control reactor consisting of a cylinder 30 cm in diameter was also established. Fresh SS was also added weekly to the control reactor following the same layering procedure used in the vermireactor. The 13-month time point was specifically selected to investigate the long-term dynamics of continuous-feeding reactors under industrial operational scenarios, and to enable comparison with our previous studies of vermicomposting sewage sludge. Previous literature mostly relies on single-dose applications (e.g., applying a single batch of waste and tracking its short-term effects), which do not capture the continuous organic matter inputs typical of industrial or full-scale vermicomposting setups, and often last no longer than 3 months. In the present study, fresh sewage sludge was progressively added to the upper layers while previously deposited material continued to undergo vermicomposting. Because vermicomposting reduces substrate mass, material accumulation within the reactor is relatively slow. Therefore, a period of 13 months was necessary to characterize the establishment, maturation, and long-term dynamics of the system under industrial operational conditions. After 13 months, the earthworm population increased to more than 20,000 individuals m^−2^. Ten subsamples were collected from each of the three different substrates: (i) initial fresh sewage sludge (S0), (ii) 13-month-aged sewage sludge without earthworms (S13), and (iii) 13-month-old vermicompost (V13) and stored at −80 °C. The plastic mesh was used to identify the layer corresponding to the SS that had been added 13 months earlier. This allowed the material sampled to be precisely dated for group comparisons. To achieve this, all the reactors were carefully excavated until the plastic mesh was located. Samples were then collected from material directly associated with the identified 13-month-old layer, ensuring that no material from the upper layers was mixed in. Ten subsamples were collected from the 13-month-old layer in each reactor and stored at −80 °C. These ten subsamples were collected from a single vermireactor to account for within-reactor heterogeneity, but they do not represent independent biological replicates of the reactor.

### 2.2. Sequencing and Bioinformatic and Statistical Analysis

DNA was extracted from 30 samples, 10 from each vermireactor layer, in a laminar flow hood to prevent contamination, using the MO-BIO PowerSoil^®^ kit (Qiagen, Hilden, Germany) according to the manufacturer’s protocol. Amplicon libraries were created using primers for the bacterial V4 hypervariable region of the 16S rRNA gene (forward GTGYCAGCMGCCGCGGTAA and reverse GGACTACNVGGGTWTCTAAT) and a fragment of the fungal ITS rRNA gene (ITSIf forward primer CTTGGTCATTTAGAGGAAGTAA and ITS2 reverse primer GCTGCGTTCTTCATCGATGC).

All bioinformatic and statistical analyses were performed in RStudio (version 2023.9.1.494) using packages phyloseq (version 1.48.0) [23], rstatix (version 0.7.3) [24], tidyverse (version 2.0.0) [25], vegan (version 2.7-5) [26], microeco (version 2.2.0) [27], patchwork (version 1.3.2) [28], picante (version 1.8.2.) [29], parallel (version 4.5.1) [30], ape (version 5,8-1) [31], nlme (version 3.1-169) [32], ggtext (version 0.1.2) [33], and ggh4x (version 0.3.1) [34]. Amplicon sequence variants (ASVs) were identified using DADA2 v 1.34.0 [35]. Sequences were processed using adapted published DADA2 pipelines for 16S (https://benjjneb.github.io/dada2/tutorial.html accessed on 1 February 2026) and ITS (https://benjjneb.github.io/dada2/ITS_workflow.html accessed on 1 February 2026). Forward and reverse reads pairs were filtered for both 16S and ITS using standard filtering parameters (maxN = 0, truncQ = 2, rm.phix = TRUE and maxEE = 2, and truncLen = c(200, 200), no truncation for ITS). ASVs were independently derived from both forward and reverse reads for each sample using run-specific error rates, after which paired reads were merged. Chimeric sequences were identified and removed per sample, and taxonomic assignment of 16S and ITS ASVs was performed using the RDP naïve Bayesian classifier against the SILVA database (version 138.2) and the UNITE database (version 10) with a bootstrap confidence threshold of 80%. ASVs that could not be classified at the Phylum level were excluded from analysis. After this analysis we retained a total of 1,577,783 sequences (50,593 ± 10,571 mean and standard deviation) distributed in 5170 ASVs for the bacteriome. For the mycobiome we retained a total of 1,099,662 sequences (36,655 ± 37,840 mean and standard deviation) distributed in 1384 ASVs. Rarefaction curves were generated, and all samples from both the bacteriome and mycobiome show appropriate diversity for analysis (Supplementary Figure S1). Sequence data were deposited in the GenBank SRA database under accession numbers PRJNA1477310 and PRJNA1477315 for bacteria and fungi, respectively.

Before differential abundance testing, ASVs present in fewer than 10% and 5% of the samples for the bacteriome and mycobiome, respectively, were eliminated. For the bacteriome, this threshold excluded 55% of the ASVs, representing approximately 1.5% of the total sequences. For the mycobiome, 64% of ASVs were excluded, representing approximately 0.62% of the total sequences. Differential abundance between treatment groups was estimated using DESeq2 at the phylum and genus levels using raw counts. The model applied a Wald test with parametric dispersion fitting and size factors estimated using the positive count geometric mean approach to address zero inflation. Because multiple pairwise Wald tests were conducted for each pairwise comparison between treatments, *p* values were further adjusted using the Benjamini–Hochberg method to correct for multiple pairwise comparisons. Results were considered statistically significant at a Benjamini–Hochberg adjusted *p*-value (Padj) ≤ 0.05.

Alpha diversity metrics were calculated at the ASV level after rarefying to the minimum sequencing depth. Observed (OTU) richness, Shannon diversity, inverse Simpson diversity, and phylogenetic diversity were used to assess microbial richness, evenness and relatedness within samples. Differences in alpha diversity across treatments (different SS and vermicompost samples) were evaluated using the Kruskal–Wallis test, followed by pairwise Wilcoxon tests.

Beta diversity was assessed at the ASV level using taxonomic and phylogenetic metrics to identify differences between groups. Bray–Curtis and Jaccard metrics were estimated to assess taxonomic differences in microbial structure, while UniFrac distances were used for phylogenetic comparisons of only 16S data. All measures were calculated using variance-stabilized transformation of count data from filtered datasets. Differences in beta diversity across treatments were calculated using permutational multivariate analysis of variance (PERMANOVA) with the adonis2 function (vegan package) using default settings. Principal coordinates analysis (PCoA) was used to identify patterns of variation across samples. We test for homogeneity of multivariate dispersion using the functions betadisper and permutest and TukeyHSD functions over results of permutest function (all in vegan package).

The abundance of human bacterial pathogens (HBPs) was estimated using methods outlined in Aira and Domínguez (2025) [36]. DADA2 function assignSpecies was used to obtain species-level taxonomic assignments. The results of ASV taxonomy were then cross-referenced with a comprehensive list of HBPs identified by Bartlett et al. [15]. Differences in HBP across treatments (different SS and vermicompost samples) were assessed using the negative binomial models in the DESeq2 package as described above.

The abundance of human fungal pathogens (HFPs) was estimated utilizing methods outlined by Aira et al. [4]. Trait information was assigned using R package FUNGuild to map ASVs to corresponding taxonomic classifications [37]. ASVs assigned to “probable” and “highly probable” categories were included in the analysis. Additionally, HFPs described in Assres et al. [38], the Eukaryotic Pathogen, Vector and Host Informatics Resource database [39], the World Health Organization Fungal Priority Pathogens List [40], and One Health: Fungal Pathogens of Humans, Animals, and Plants report were included [41]. The final list of HFP included 40 genera and 137 species. Differences in HFPs across treatments (different SS and vermicompost samples) were assessed using the negative binomial models in the DESeq2 package.

Bacterial functional profiles were predicted using the Phylogenetic Investigation of Communities by Reconstruction of Unobserved States (PICRUSt2), following the standard pipeline and the KEGGREST package (version 1.52.2) [42,43]. Using methods outlined by Domínguez et al. [13], predicted metabolic pathways related to amino acid biosynthesis, furfural and bisphenol degradation, antibiotic synthesis, antibiotic resistance, plant hormone synthesis and nitrogen metabolism were compared at each layer, based on their relevance to the main biological processes associated with vermicomposting and to facilitate comparison with previous studies. Differences in abundance across predicted functional categories were evaluated using the Kruskal–Wallis and Wilcoxon tests. Principal coordinates analysis (PCoA) was also performed using the predicted KEGG functional profiles to assess overall differences in functional composition among treatments. Similarly, fungal ecological trophic modes were assessed at treatment (different SS and vermicompost samples) following assignments in the FUNguild database [37]. Differences in trophic mode abundances were evaluated using Kruskal–Wallis tests and Wilcoxon tests.

## 3. Results

In the bacteriome, S0 had 860 unique ASVs, S13 had 860, and V13 had 1781. A total of 415 ASVs were shared across all layers in the bacteriome. In the mycobiome, S0 had 612 unique ASVs, S13 had 156, and V13 had 166. Ninety ASVs were shared across all groups (Supplementary Figure S2).

### 3.1. Taxonomic Composition and Diversity of Microbiomes

The main phyla in the bacteriome were Pseudomonadota that dominated at each stage (S0: 59.22%, S13: 41.86%, V13: 37.85%), followed by Actinomycetota (S0: 18.52%, S13: 31.48%, V13: 25.58%, Figure 1). At the genus level, a mixture of genera dominated at different abundance across treatments (i.e., different treatments and ages), the most prominent including *Mycobacterium*, unclassified *PeM15*, and *Rhodanobacter* (Figure 1). Pairwise comparisons identified up to 29 phyla showing statistically significant (*p*-adj ≤ 0.05) differences in mean relative abundance in S0 vs. V13, S13 vs. V13, and S0 vs. S13 (Supplementary Table S1). Up to 465 genera showed statistically significant (*p*-adj ≤ 0.05) differences in mean abundance for the same three pairwise comparisons (Supplementary Table S2).

The mycobiome was dominated by the phyla Ascomycota (S0: 11.97%, S13: 57.19%, V13: 89.68%) and Basidiomycota (S0: 87.93%, S13: 42.72%, V13: 9.78%), with a clear shift from Basidiomycota towards Ascomycota across both the control and vermicomposted samples (Figure 1). The most abundant genera include *Apiotrichum*, *Cephaliphora*, and *Scedosporium* with dominance varying across sludge and vermicompost and time. Up to four phyla showed statistically significant differences in relative mean abundance across groups (S0 vs. V13, S13 vs. V13, and S0 vs. S13) (Supplementary Table S3). At the genus level, up to 58 genera showed statistically significant differences (*p*-adj ≤ 0.05) in mean abundance for the same three pairwise comparisons (Supplementary Table S4).

Alpha diversity measures of richness and evenness were estimated through observed richness, Shannon diversity, and Inverse Simpson diversity. Across all measures, bacterial alpha diversity increased progressively with time, from S0 through S13, with V13 showing the highest values. All comparisons between groups were statistically significant (*p*-adj ≤ 0.05), apart from ASV richness for S0 vs. S13 (Figure 2, Supplementary Figure S3). In contrast to the bacteriome, fungal richness decreased across treatments, while Shannon and Inverse Simpson increased (Figure 2). Almost all pairwise comparisons show statistically significant differences (*p*-adj ≤ 0.05) between groups and measures, except S13 vs. V13.

PCoA of beta diversity for the bacteriome showed clear separation of S0, S13, and V13 across all estimated distances (Bray–Curtis and Jaccard, Figure 3; weighted and unweighted Unifrac, Supplementary Figure S4). PERMANOVA analyses showed statistically significant (*p*-adj ≤ 0.01) differences in community structure across all comparisons, with variance explained (R^2^) ranging from 54.66% to 78.39%. Tests for homogeneity of multivariate dispersion indicated significant variance heterogeneity for Bray–Curtis (PERMDISP F = 4.86, *p* = 0.008) and Jaccard (F = 4.21, *p* = 0.010) metrics, statistically driven by pairwise differences between vermicompost (V13) and initial sludge (S0; Tukey’s HSD *p* = 0.020 and *p* = 0.042, respectively). In contrast, UniFrac metrics exhibited homogeneous dispersions across treatments (*p* > 0.48), and ordination plots confirmed that clear centroid separation, rather than dispersion artifacts, remains the primary driver of the observed PERMANOVA differences.

Beta diversity analysis of the mycobiome showed distinct clustering across all groups for both estimated distances (Bray–Curtis and Jaccard). PERMANOVA analyses confirmed statistically significant (*p*-adj ≤ 0.01) differences in community structure across all comparisons, with variance (R^2^) ranging from 16.28% to 48.97% (Figure 3). Multivariate dispersion tests revealed significant heterogeneity among groups for both Bray–Curtis (PERMDISP F = 5.99, *p* = 0.008) and Jaccard (F = 10.63, *p* = 0.001). Post hoc Tukey HSD comparisons indicated that dispersion differences were statistically driven by initial sludge (S0) having lower multivariate dispersion compared to both 13-month control sludge (S13; *p* ≤ 0.008) and vermicompost (V13; *p* ≤ 0.037). Nevertheless, clear visual separation of centroids across ordination space indicates that treatment differences remain the primary determinant of community variation.

Twenty-seven human bacterial pathogens were identified across all treatments, each with mean read counts ranging from 0.03 (*Bacteroides stercoris*, *Parabacteroides distasonis*) to 70.7 (*Turicibacter sanguinis*). S0 contained 14 HBPs, S13 contained 9, and V13 contained 17 (Figure 4). Up to nine distinct HBPs showed statistically significant (*p*-adj ≤ 0.05) changes in abundance between groups: one in S13 vs. V13, seven in S13 vs. S0, and eight in V13 vs. S0 (Supplementary Table S5).

Fifty-three human fungal pathogens were identified in the mycobiome; S0 contained 41, S13 contained 20, and V13 contained 23 (Figure 4). Mean microbial abundance ranged from 0.01 (unclassified *Aspergillus* and *Penicillium multicolor*) to 280.11 (*Cystobasidium slooffiae*) reads (Figure 4). Up to ten distinct HFPs showed statistically significant (*p*-adj ≤ 0.05) changes in abundance between groups: six in S13 vs. V13, four in S0 vs. S13, and 16 in S0 vs. V13 (Supplementary Table S6).

### 3.2. Functional Diversity of Microbiomes

The functional potential of the bacteriome was evaluated across compost and vermicompost groups using PICRUSt2. Genes associated with predicted antibiotic resistance and furfural degradation decreased in the S13 and V13 groups, while genes associated with predicted bisphenol degradation, amino acid biosynthesis, antibiotic synthesis, and nitrogen metabolism increased in the same groups (Figure 5). Statistically significant (*p*-adj ≤ 0.05) differences in abundance were identified across comparisons of S0 vs. S13 and S0 vs. V13 for most predicted pathways; S13 vs. V13 showed significant differences only for predicted antibiotic resistance and furfural degradation. Predicted plant hormone synthesis and bisphenol degradation were not significant across any comparison.

To further investigate the predicted functional profiles, a PCoA of KEGG pathways was performed using Bray–Curtis and Jaccard distances. Predicted functional profiles showed clear separation among the S0, S13, and V13 groups (Supplementary Figure S5). Pairwise PERMANOVA tests indicated statistically significant differences between all pairwise comparisons (*p*-adj < 0.001). These results indicate substantial shifts in predicted functional composition during sludge maturation and vermicompost processing.

Seven fungal trophic modes were compared across treatments. Increases in fungal richness were observed in Pathotroph–Saprotroph–Symbiotroph, Saprotroph, Saprotroph–Symbiotroph, and Pathotrophic modes from S0 to S13 and V13 (Figure 6). Decreases were observed in Pathotroph-Symbiotroph and Symbiotroph modes from S0 to S13 and V13. Pairwise comparisons showed statistically significant (*p*-adj < 0.05) differences in most trophic modes across groups; S0 vs. V13 showed significant differences in all trophic modes except Saprotroph–Symbiotroph, while S0 vs. S13 showed significant differences in all modes except Pathotroph–Saprotrophic–Symbiotroph and Saprotroph–Symbiotroph. S13 vs. V13 showed significant differences in Pathotroph–Saprotrop–Symbiotroph and Saprotroph only.

## 4. Discussion

Previous studies have shown that vermicomposting substantially alters the microbial ecology of sewage sludge. Researchers have characterized bacterial succession during vermicomposting and demonstrated that earthworm activity drives changes in microbiome composition and function [4,13]. Subsequent studies expanded these observations to fungal communities and reported reductions in pathogen loads and antibiotic resistance determinants during vermicomposting [4,16]. Consistent with previous work, this study revealed extensive restructuring of both bacterial and fungal communities, shifts in predicted functional profiles, and changes in the abundance and diversity of human-associated pathogens. By simultaneously evaluating bacterial and fungal communities during both sludge maturation and vermicomposting, the study provides a more comprehensive view of microbial succession and enables direct comparison between vermicomposting and natural sludge maturation. This distinction is particularly important because it allows microbial changes associated with vermicomposting to be assessed against those occurring during maturation alone under comparable environmental conditions.

Results from taxonomic composition, diversity analyses, and functional predictions indicate that vermicomposting seems to exert microbial effects that differ from those associated with passive sludge maturation alone. These findings are consistent with the hypothesis that earthworm-mediated processing modifies community assembly through mechanisms that extend beyond natural aging processes, including gut-associated processes (GAPs) and cast-associated processes (CAPs), both of which have been recognized as major determinants of microbial turnover in decomposing organic substrates [12,44,45].

At the bacterial level, both maturation and vermicomposting were associated with a decrease in Pseudomonadota, a phylum frequently linked to nutrient-rich environments and ARG reservoirs [46,47]. Despite this decline, Pseudomonadota remained abundant across all stages, consistent with reports that sewage sludge can act as a persistent source of this taxon [48]. The gradual reduction in this group may reflect increasing substrate stabilization, as its abundance has been associated with compost immaturity [49]. In the maturation-only control, shifts toward Actinomycetota and away from Bacillota were observed, consistent with the enrichment of oligotrophic taxa adapted to nutrient-depleted environments [50]. However, this trajectory was not fully mirrored in vermicompost samples, suggesting that earthworm activity may disrupt conventional successional patterns and promote distinct community assembly predicted pathways. Substantial turnover at the genus level further supports the idea that vermicomposting may lead to strong microbial reorganization, likely through selective ingestion, digestion, and redistribution of microorganisms [4,13].

Fungal communities exhibited comparable but more pronounced shifts. Across all treatments, Basidiomycota and Ascomycota dominated, consistent with their central roles in decomposition and nutrient cycling [51]. Both maturation and vermicomposting showed a shift toward increased relative abundance of Ascomycota; however, this transition was more pronounced under vermicomposting. This suggests that earthworm activity may amplify natural successional processes, potentially by favoring fast-growing or disturbance-tolerant fungal taxa.

Patterns of alpha diversity further differentiated the two processes. Bacterial diversity increased progressively across all stages, with the highest values observed in V13, indicating continuous diversification and niche differentiation. In contrast, fungal richness decreased while evenness increased, suggesting selective filtering toward more evenly distributed communities. These findings indicate that vermicomposting both may reshape and intensify the natural maturation process rather than functioning as an entirely independent pathway. Beta diversity analyses seem to support this interpretation, with S13 and V13 showing distinct microbial community structures after the same 13-month period.

Human-associated bacterial and fungal pathogens were detected throughout the study, although both sludge maturation and vermicomposting altered their composition, abundance, and dynamics, with vermicomposting producing more pronounced changes in fungal pathogen communities than maturation alone. However, the generally low read counts observed for many taxa indicate that these findings should be interpreted cautiously, as detection does not necessarily indicate viability or pathogenic risk [52,53]. Further studies incorporating viability assessments would help clarify the implications of these compositional changes for biosolids safety.

Functional predictions also revealed significant shifts in multiple pathways. The observed decrease in predicted antibiotic resistance-related genes supports the role of vermicomposting in sewage sludge stabilization and potential detoxification [4,54]. At the same time, increases in predicted pathways associated with nitrogen metabolism, amino acid biosynthesis, and xenobiotic degradation (e.g., bisphenols) may suggest metabolic versatility. In the mycobiome, shifts toward saprotrophic and mixed trophic modes further indicate enrichment of decomposer-driven communities associated with nutrient cycling and carbon turnover [55,56].

Previous vermicomposting studies have shown that, after three months of processing, vermicomposts exhibit significantly lower pathogen loads and reduced abundance of antibiotic resistance genes (ARGs), as determined either by qPCR [12,16] or amplicon-based metagenomics [54]. Because these studies used sewage sludge from different wastewater treatment plants, it can be inferred that, although the magnitude of pathogen and ARG reductions may vary among sludge types, vermicomposting consistently promotes the sanitization of sewage sludge. However, these experiments were conducted using a single initial dose of sewage sludge, with the material applied only once and changes assessed after three months of vermicomposting. That experimental design differs from that of vertically operated, continuously fed vermireactors like the one used in the present study, in which fresh sewage sludge is periodically added to the upper layers as previously deposited material undergoes processing. In these systems, the moisture associated with newly added sewage sludge may facilitate the downward leaching of partially processed material into deeper layers that have already undergone vermicomposting, potentially resulting in the reintroduction or redistribution of microorganisms and contaminants. Such a process has been previously described in these vermicomposting systems [12,57]. Therefore, the significant increases in the abundance of several pathogens observed in our study may be partly explained by the continuous addition of fresh sewage sludge and its subsequent leaching through the vermireactor, which was fed weekly throughout the study. Interestingly, however, the effects of vermicomposting on predicted ARGs in our study were consistent with those reported for single-dose vermicomposting systems, with an overall reduction in ARG abundance. This pattern was observed both when ARGs were directly quantified by qPCR [16] and when their abundance was inferred using PICRUSt2 [54]. These findings suggest that ARG reduction occurred during earthworm gut transit [16,54], potentially limiting the reintroduction of ARGs associated with newly added sewage sludge and making this process less susceptible to the effects of continuous feeding vermicomposting systems. The effectiveness of this mechanism may therefore depend on earthworm density, which could determine the proportion of newly added sewage sludge that undergoes earthworm gut transit before leaching into previously processed layers.

An important aspect of the present study is also the use of an industrial-scale vermicomposting system. Such systems are rarely investigated using high-resolution microbial community analyses due to the logistical and operational challenges associated with these facilities. As a result, much of the current understanding is derived from laboratory-scale experiments. One of the primary objectives of this work was to evaluate whether microbial patterns previously observed under controlled experimental conditions could also be detected in real operational systems. Our findings, therefore, extend beyond previous observations by showing that bacterial and fungal community restructuring are detectable at an industrial scale. In doing so, this study provides a detailed characterization of bacterial and fungal communities associated with industrial sewage sludge processing systems, generating information that can guide future hypothesis-driven studies.

Additionally, several metrics of microbial composition, diversity, community structure, and predicted function differed at 13 months and may continue to develop beyond this time point. This further suggests that microbial succession remains active over extended periods in continuous-feeding systems. Because the present study did not extend beyond 13 months, it remains unclear whether the observed communities had reached a stable state. Longer-term monitoring would help determine whether these successional trajectories eventually stabilize and establish the time required for vermicompost to reach maturity.

Several limitations should be considered when interpreting the findings of the present study. First, this study included only one control reactor and one vermireactor. Consequently, the subsamples analyzed within each reactor represent technical replicates rather than independent biological replicates. As a result, statistical tests based on these subsamples should be interpreted with caution because they do not provide true replication at the reactor level and may overestimate the strength of evidence for treatment effects. Therefore, the observed differences should be considered exploratory and specific to the reactors studied, and the results are not readily generalizable to other vermicomposting systems. This experimental design also limits the suitability of this dataset for taxonomic co-occurrence network analyses. Second, the lack of physicochemical characterization of the sewage sludge and vermicomposting samples limits the ability to directly relate changes in microbial communities to specific environmental drivers, thereby constraining interpretation of the observed patterns. Third, functional profiles were predicted using 16S abundances and PICRUSt2; future work should prioritize higher-resolution data types, such as shotgun metagenomics, to enable robust inferencing of functional profiles and co-occurrence networks. Moreover, the number of counts assigned to individual predicted KEGG sub-pathways within functional categories (e.g., antibiotic resistance and antibiotic biosynthesis) was generally low, preventing detailed and robust statistical comparisons. Shotgun metagenomic approaches could provide greater functional resolution and enable more comprehensive investigation of these pathways.

## 5. Conclusions

Using control one pilot-scale reactor and one pilot-scale vermireactor, this study shows that vermicomposting significantly reshapes the bacterial and fungal microbiomes of sewage sludge through earthworm-mediated processes. These changes include shifts in taxonomic composition, community structure, and diversity, as well as a restructuring of predicted functional profiles toward enhanced decomposition, nutrient cycling, and reduced antibiotic resistance. Our results show that vermicomposting follows a distinct trajectory compared to natural sludge maturation. Importantly, vermicompost maturation is a dynamic phase of ongoing microbial succession, during which both bacteriome and mycobiome communities seem to increase in diversity and functional specialization. The observed enrichment of predicted pathways associated with nutrient cycling and pollutant degradation highlights the potential benefits of extended maturation periods for improving vermicompost quality. The reduction in predicted antibiotic resistance-related pathways underscores the potential of vermicomposting as a strategy for sludge stabilization and risk mitigation. Both sludge maturation and vermicomposting reduced the richness of human-associated fungal pathogens and significantly altered pathogen community composition, supporting their use as treatment strategies prior to land application. However, because some human-associated pathogens remained detectable after treatment and viability was not assessed, composted and vermicomposted sewage sludge should be applied in accordance with existing biosolids regulations. Additional studies evaluating pathogen viability would further strengthen assessments of their suitability for agricultural use.

## Figures and Tables

**Figure 1:**
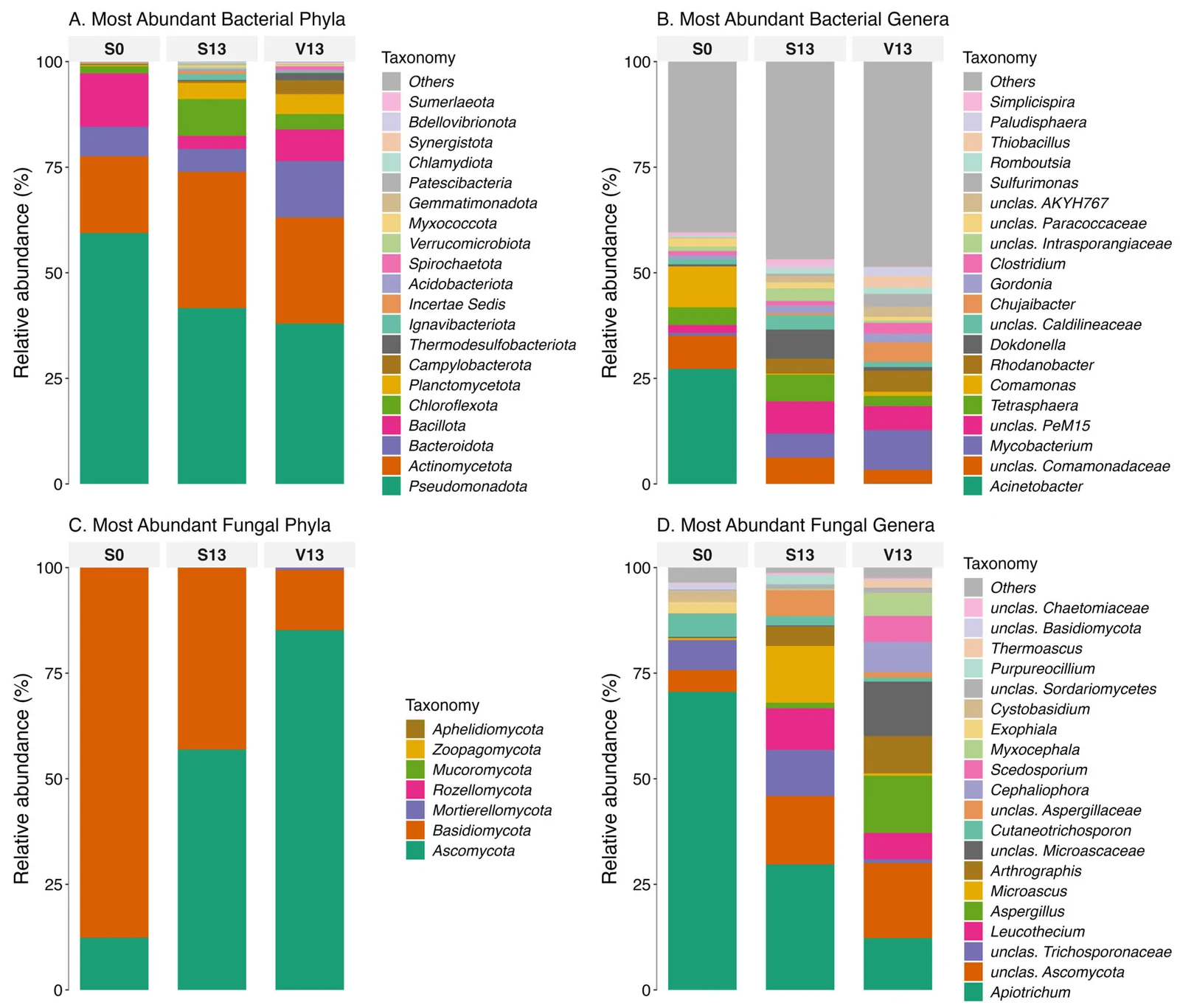
Relative abundance of most abundant bacterial and fungal phyla and genera in sewage sludge and vermicompost samples of different ages [0 (S0) and 13 (S13 and V13) months)].

**Figure 2:**
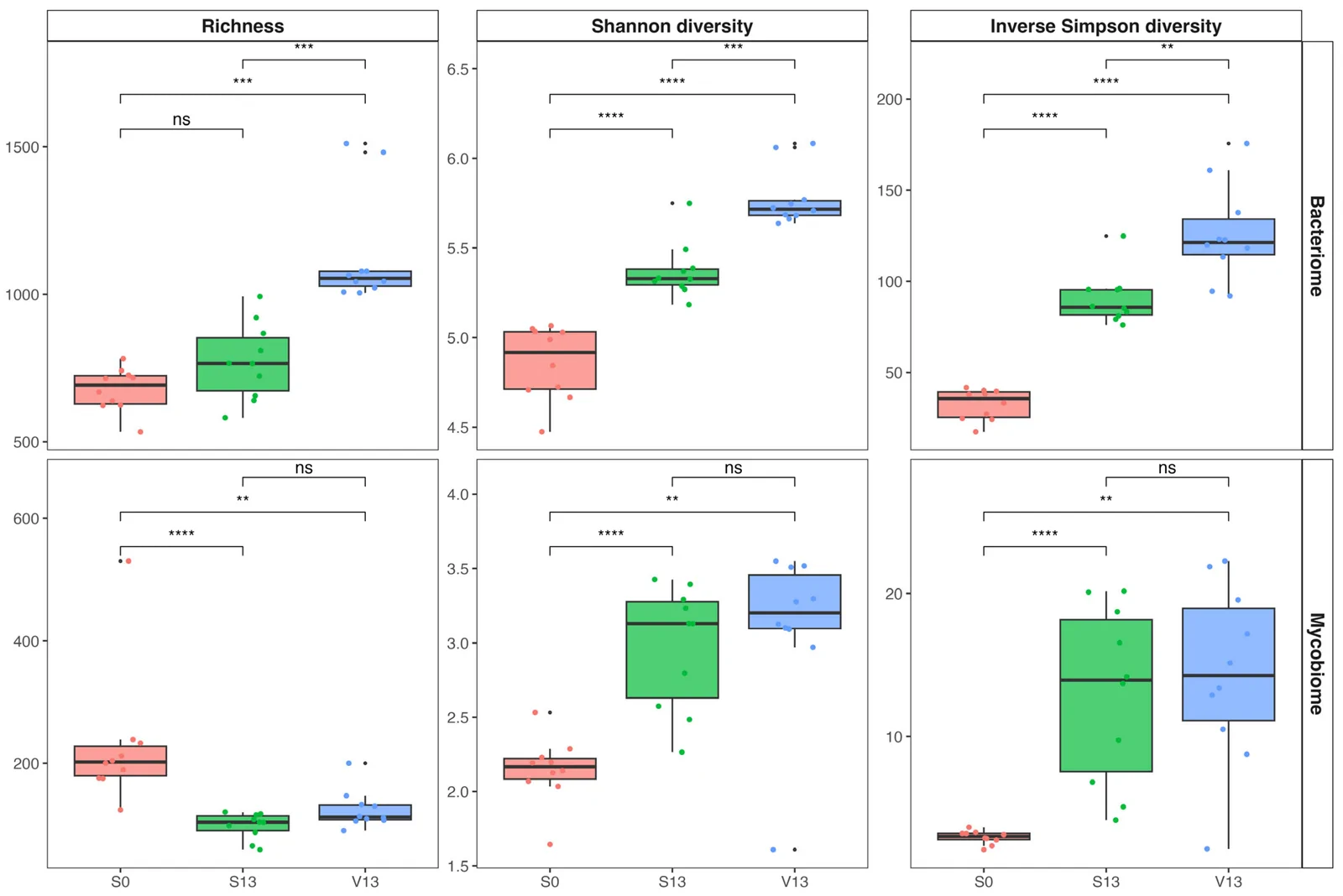
Changes in bacterial and fungal alpha diversity in sewage sludge and vermicompost samples of different ages [0 (S0) and 13 (S13 and V13) months)]. ** *p* < 0.01, *** *p* < 0.001, and **** *p* < 0.0001.

**Figure 3:**
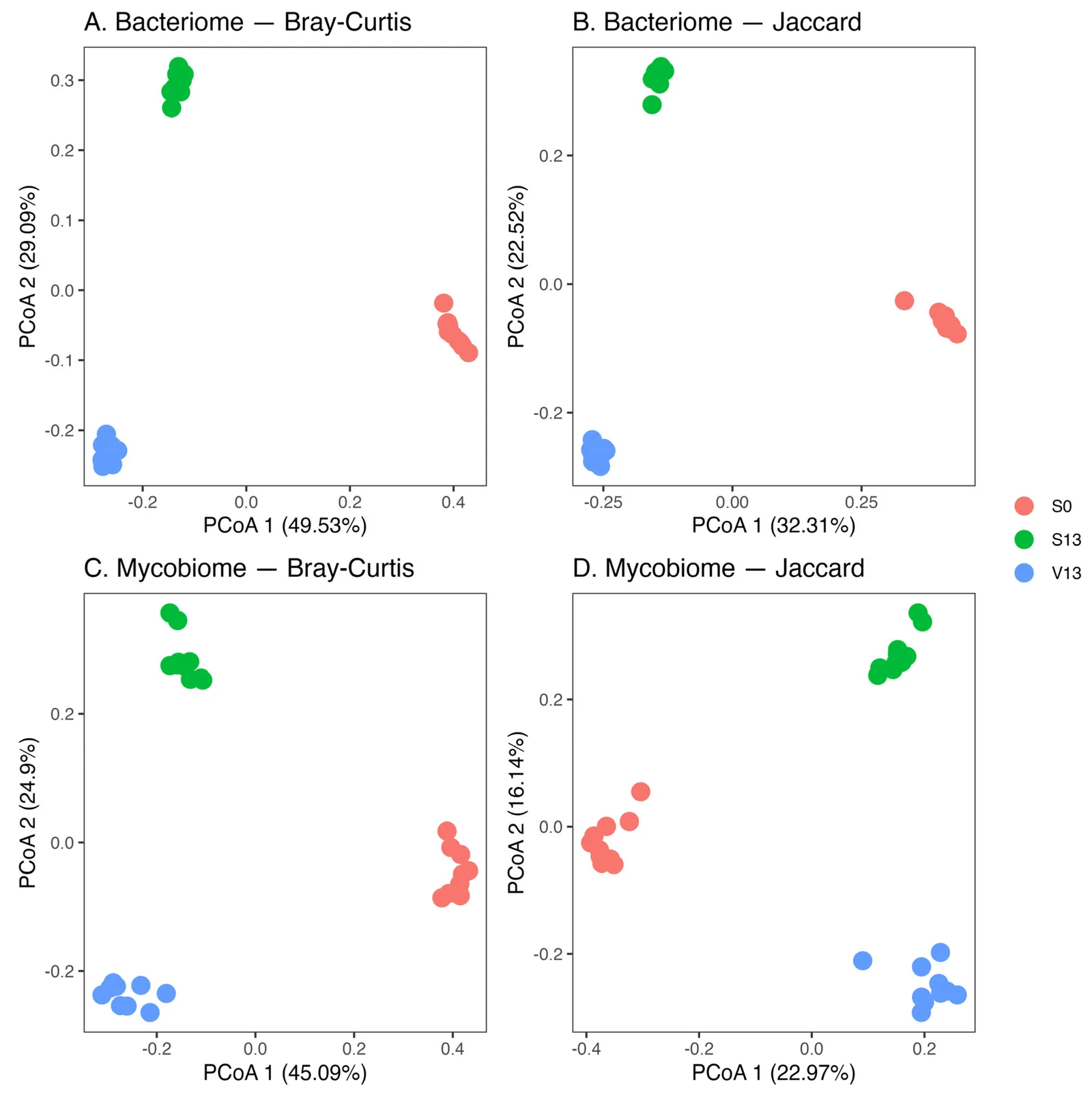
Changes in beta diversity in sewage sludge and vermicompost samples of different ages [0 (S0) and 13 (S13 and V13) months)].

**Figure 4:**
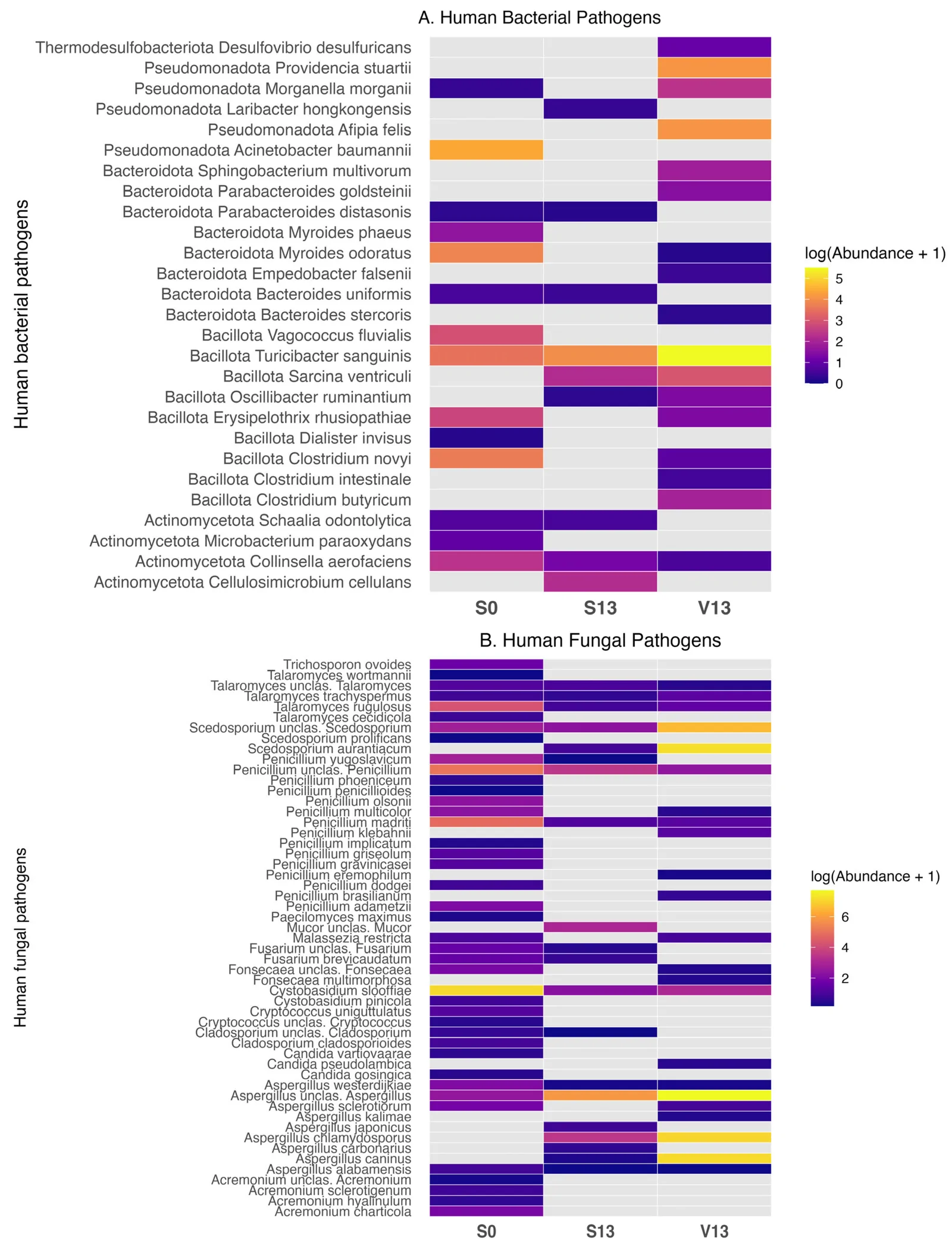
Changes in the abundance of human bacterial and fungal pathogens in sewage sludge and vermicompost samples of different ages [0 (S0) and 13 (S13 and V13) months)].

**Figure 5:**
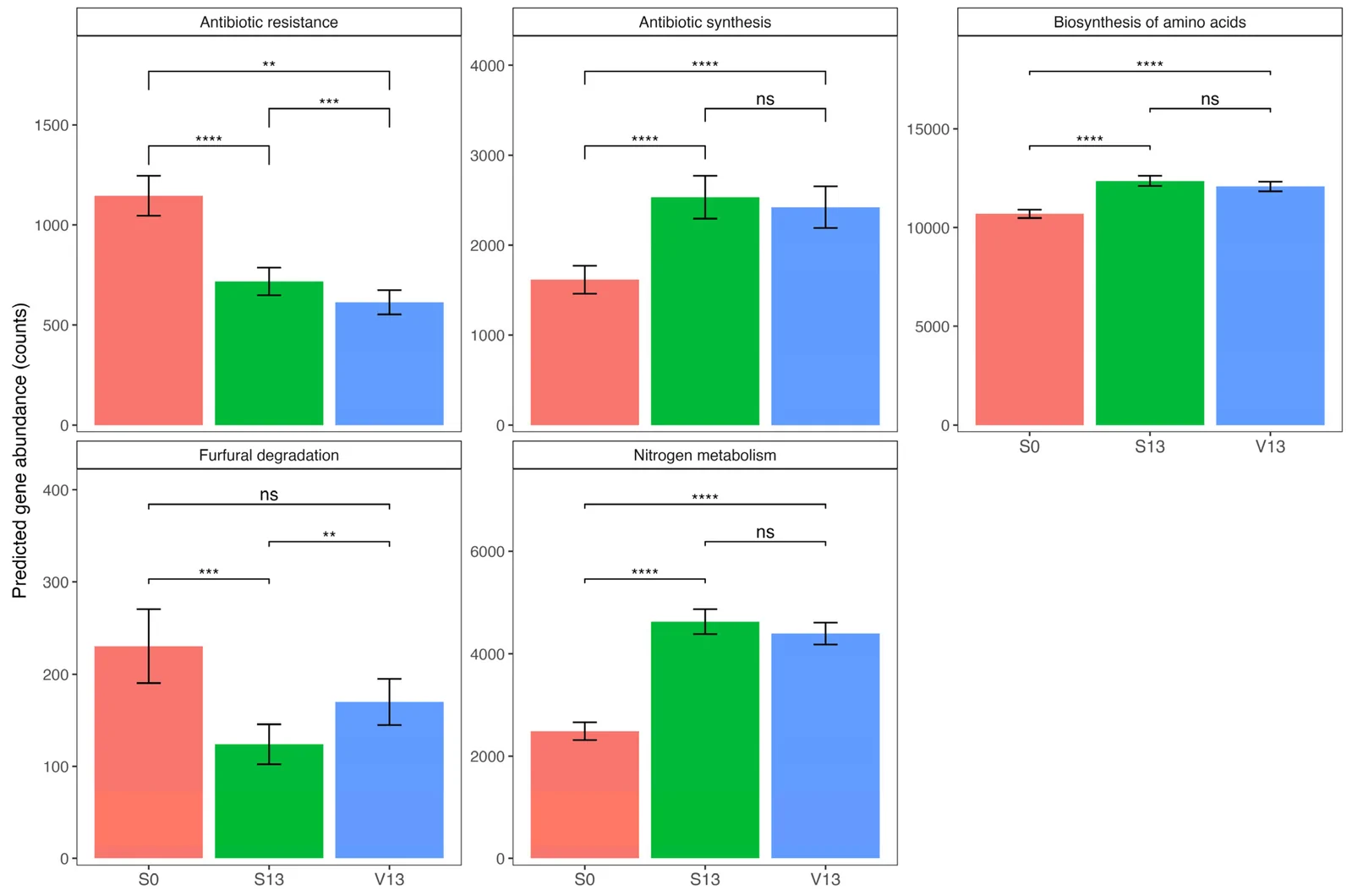
Changes in the abundance of selected bacterial predicted KEGG pathways in sewage sludge and vermicompost samples of different ages [0 (S0) and 13 (S13 and V13) months)]. ** *p* < 0.01, *** *p* < 0.001, and **** *p* < 0.0001.

**Figure 6:**
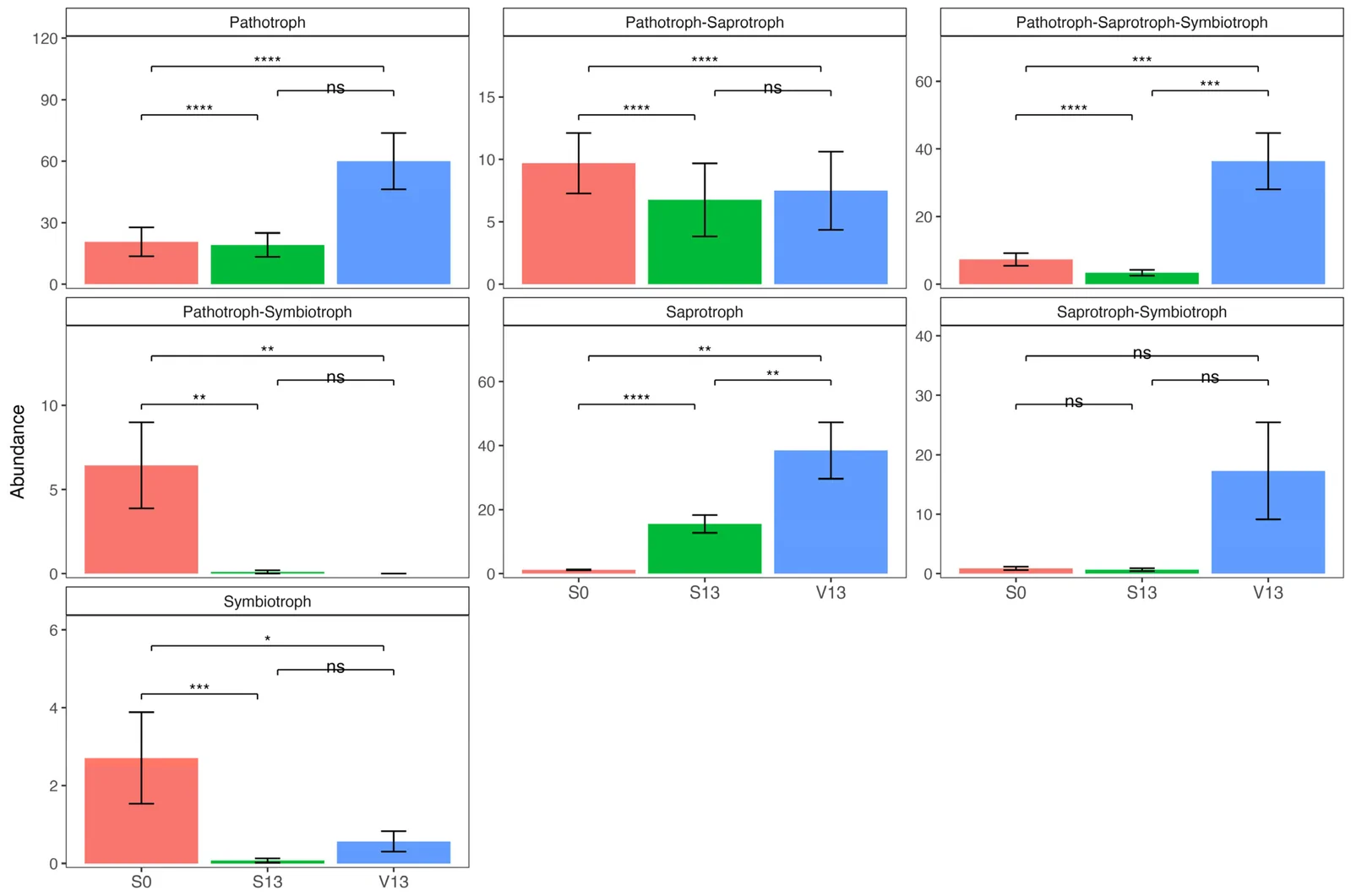
Changes in the abundance of fungal trophic modes in sewage sludge and vermicompost samples of different ages [0 (S0) and 13 (S13 and V13) months)]. Asterisks indicate statistically significant differences: * *p* < 0.05, ** *p* < 0.01, *** *p* < 0.001, and **** *p* < 0.0001.

## Data Availability

All code and data used in this study is available at https://github.com/iberdecio3-gw/16S-ITS-Vermicompost-Analysis, accessed on 1 February 2026.

## Supplementary Materials

The following supporting information can be downloaded at: https://www.mdpi.com/article/10.3390/biotech15040083/s1, Figures: Supplementary Figure S1. Rarefaction curves showing ASV richness for (A) 16S rRNA gene sequences (bacteriome) and (B) ITS sequences (mycobiome), Supplementary Figure S2. UpSet plots showing shared and unique amplicon sequence variants (ASVs) for (A) 16S rRNA gene sequences (bacteriome) and (B) ITS sequences (mycobiome), Supplementary Figure S3. Bacterial alpha diversity measured by Faith’s Phylogenetic Diversity across groups, Supplementary Figure S4. Principal coordinates analysis (PCoA) of bacterial beta diversity based on unweighted and weighted UniFrac distances, Supplementary Figure S5. Principal coordinate analysis (PCoA) of PICRUSt2-predicted bacterial functional profiles based on Bray–Curtis and Jaccard distances across S0, S13, and V13 groups. Tables: Supplementary Table S1. DESeq2 results for bacterial phyla exhibiting significant differences in relative abundance across pairwise comparisons among sludge maturation and vermicomposting treatments, Supplementary Table S2. DESeq2 results for bacterial genera exhibiting significant differences in relative abundance across pairwise comparisons among sludge maturation and vermicomposting treatments, Supplementary Table S3. Differentially abundant human bacterial pathogens identified by DESeq2 across pairwise treatment comparisons, Supplementary Table S4. DESeq2 results for fungal genera exhibiting significant differences in relative abundance across pairwise comparisons among sludge maturation and vermicomposting treatments, Supplementary Table S5. Differentially abundant human bacterial pathogens identified by DESeq2 across pairwise treatment comparisons, Supplementary Table S6. Differentially abundant human fungal pathogens identified by DESeq2 across pairwise treatment comparisons.
