# Supplementary material for: Vermicomposting Reshapes Microbial Communities and Functional Potential in Sewage Sludge: Insights from 16S and ITS Metabarcoding
Source: BioTech (Basel). 2026 Sep 25;15(4):83. doi: 10.3390/biotech15040083 (PMC13702783; doi:10.3390/biotech15040083)
Supplement: Supplementary file 1 [file biotech-15-00083-s001.zip › Supplementary Figure S2.pdf]

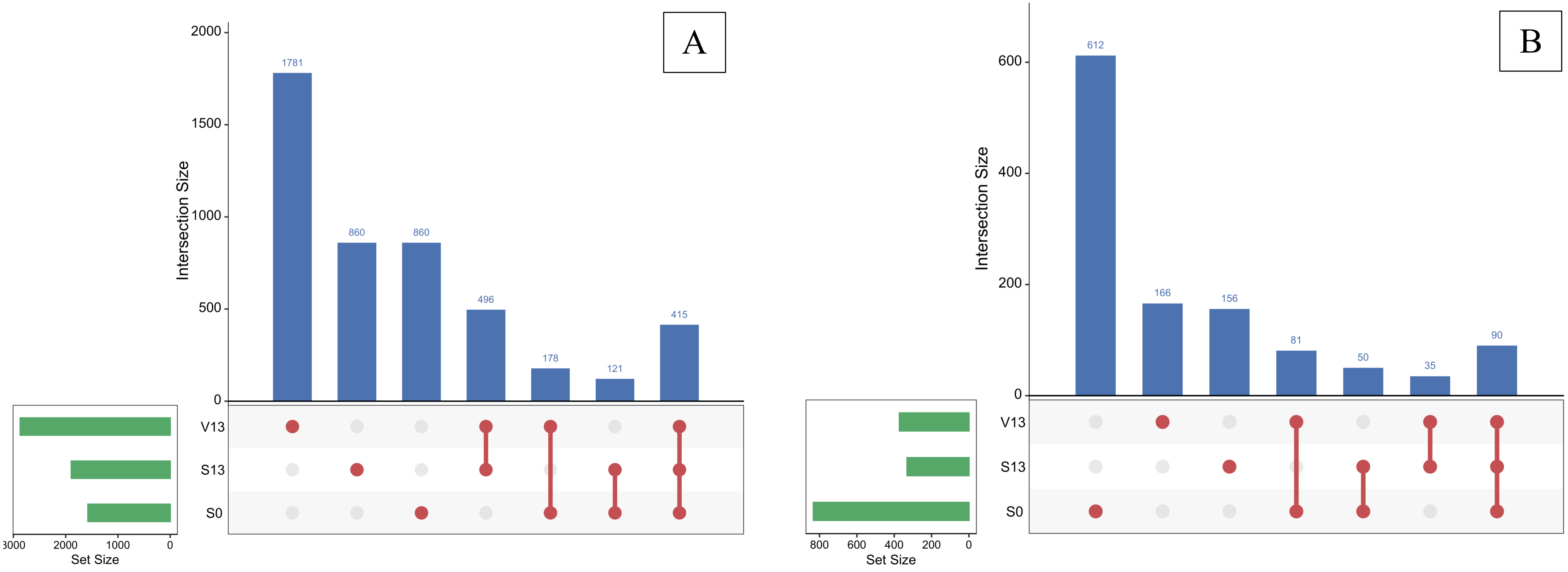

Supplementary Figure S2. UpSet plots showing shared and unique amplicon sequence variants (ASVs) for (A) 16S rRNA gene sequences (bacteriome) and (B) ITS sequences (mycobiome).
