# Supplementary material for: Vermicomposting Reshapes Microbial Communities and Functional Potential in Sewage Sludge: Insights from 16S and ITS Metabarcoding
Source: BioTech (Basel). 2026 Sep 25;15(4):83. doi: 10.3390/biotech15040083 (PMC13702783; doi:10.3390/biotech15040083)
Supplement: Supplementary file 1 [file biotech-15-00083-s001.zip › Supplementary Figure S4.pdf]

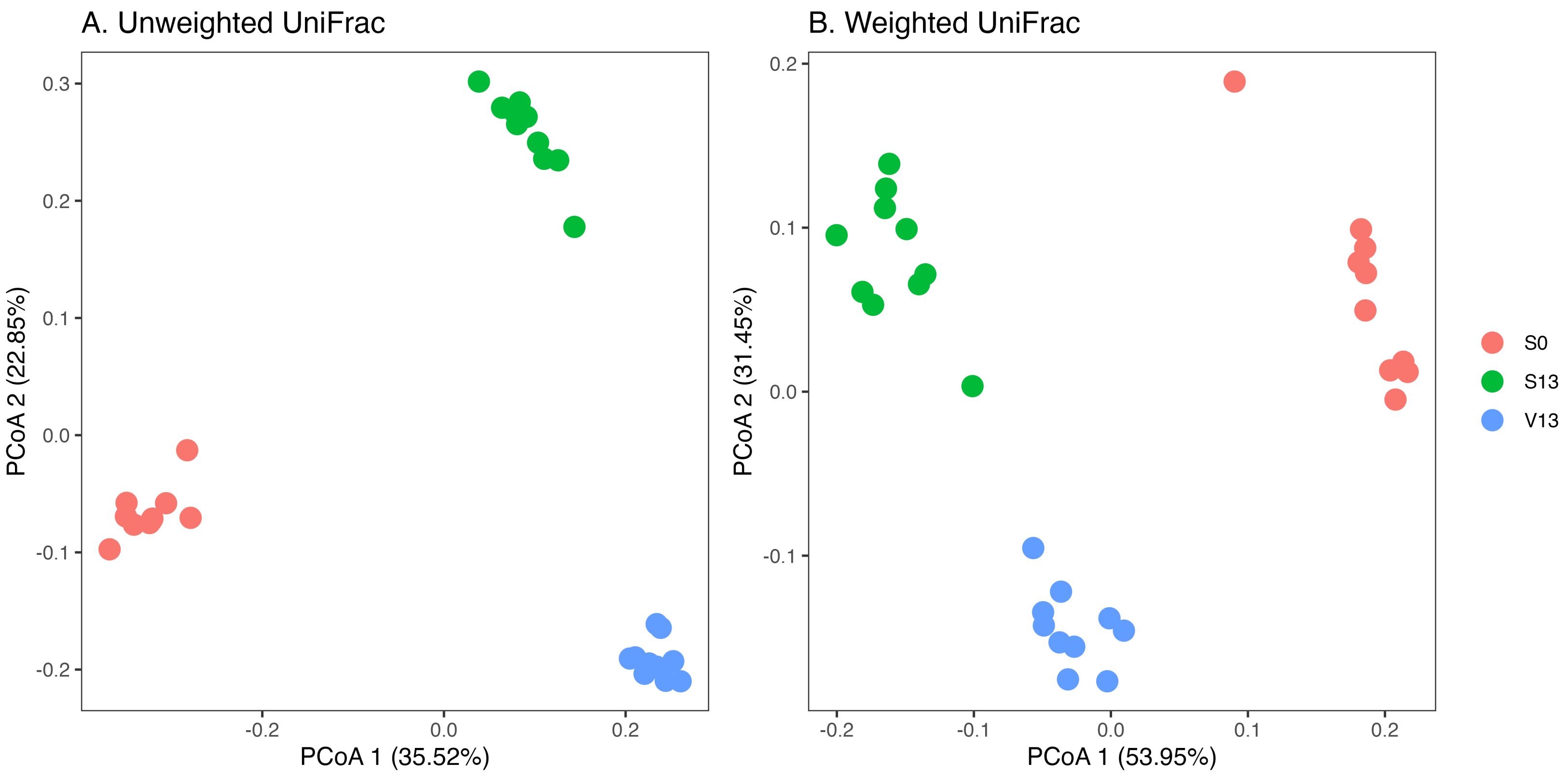

Supplementary Figure S4. Principal coordinates analysis (PCoA) of bacterial beta diversity based on unweighted and weighted UniFrac distances.
